# Supplementary material for: Supra-biological performance of immobilized enzymes enabled by chaperone-like specific non-covalent interactions
Source: Nat Commun. 2024 Mar 14;15:2299. doi: 10.1038/s41467-024-46719-5 (PMC10940687; doi:10.1038/s41467-024-46719-5)
Supplement: Supplementary file 1 — Supplementary Information [file 41467_2024_46719_MOESM1_ESM.pdf]

Supplementary Information for

Supra-Biological Performance of Immobilized Enzymes Enabled by Chaperone-like Specific Non-Covalent Interactions

Héctor Sánchez-Morán, Joel L. Kaar, and Daniel K. Schwartz**

Department of Chemical and Biological Engineering, University of Colorado, Campus Box 596, Boulder, CO, 80309, USA

*Corresponding Authors:

Joel L. Kaar. Email: joel.kaar@colorado.edu

Daniel K. Schwartz. Email: daniel.schwartz@colorado.edu

This PDF file includes:
Supplementary Figures 1 to 20
Supplementary Tables 1 to 10
Supplementary Methods

Supplementary Figures

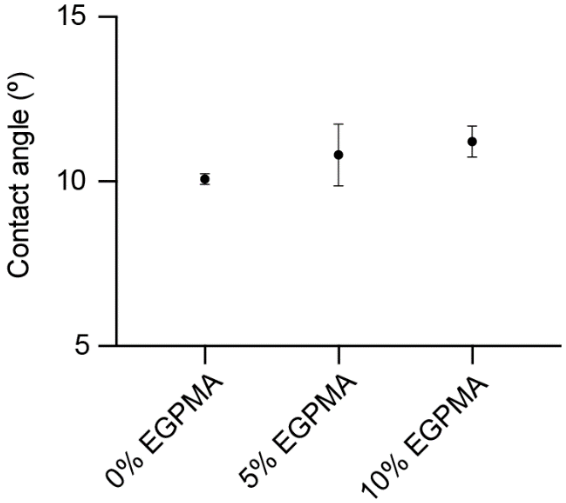

Supplementary Figure 1. Water contact angles of flat surfaces functionalized with SBMA/EGPMA polymer brushes, measured by the sessile drop method, showing the similar hydrophilicity of 0 – 10% EGPMA surfaces. Error bars represent the standard error of the mean for five technical replicates (n = 5).

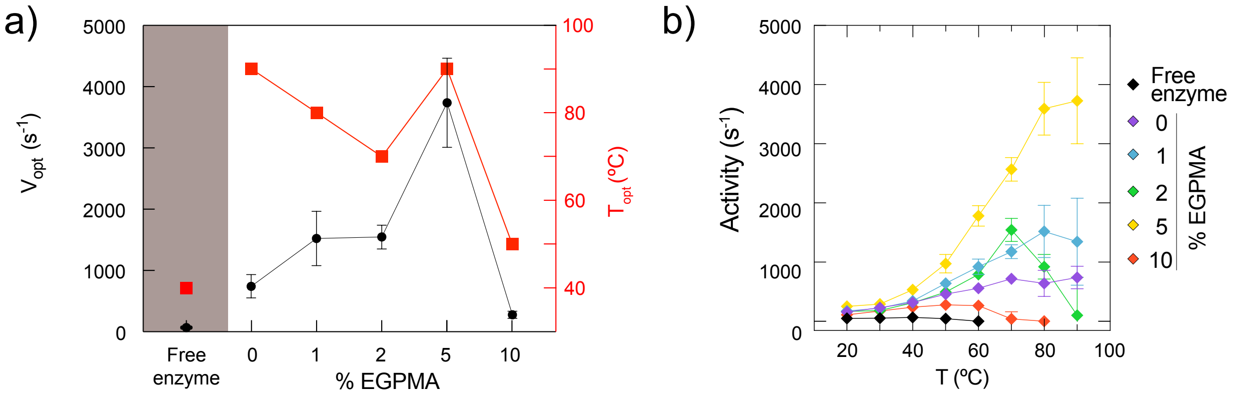

Supplementary Figure 2. a) Optimal activity (V_{opt}) and temperature of optimal activity (T_{opt}) of immobilized LipA extracted from **Fig. 2a** as a function of polymer brush composition. The data points in the shaded region correspond to V_{opt} and T_{opt} values for free LipA. Error bars represent the standard error of the mean for three technical replicates (n = 3). b) Representation of data from **Fig. 2a** with activity plotted on a linear scale. Error bars represent the standard error of the mean for three technical replicates (n = 3).

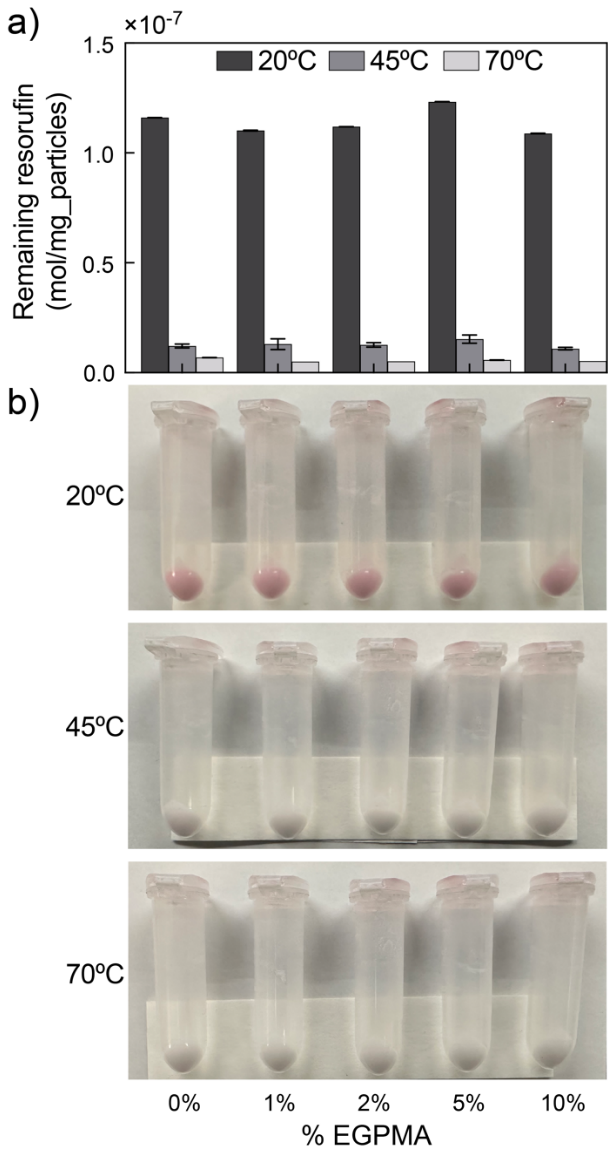

Supplementary Figure 3. Resorufin adsorption on SBMA/EGPMA supports as a function of temperature. **a)** Remaining resorufin fluorescence when adsorbed on 0 – 10% EGPMA-containing particles after 3 rounds of washing with buffer at 20, 45 or 70 ºC. Previously, particles had been incubated with 50 μM resorufin for 20 minutes at room temperature. Resorufin adsorption was independent of EGPMA content, and negligible at high temperatures. Error bars represent the standard error of the mean for three technical replicates (n = 3). **b)** Pictures of particles after washing with buffer at 20, 45 or 70ºC. The observed pink color corresponds to adsorbed resorufin on particles. For this experiment, particles without GMA were used to avoid covalent coupling of resorufin to the epoxide moiety of GMA.

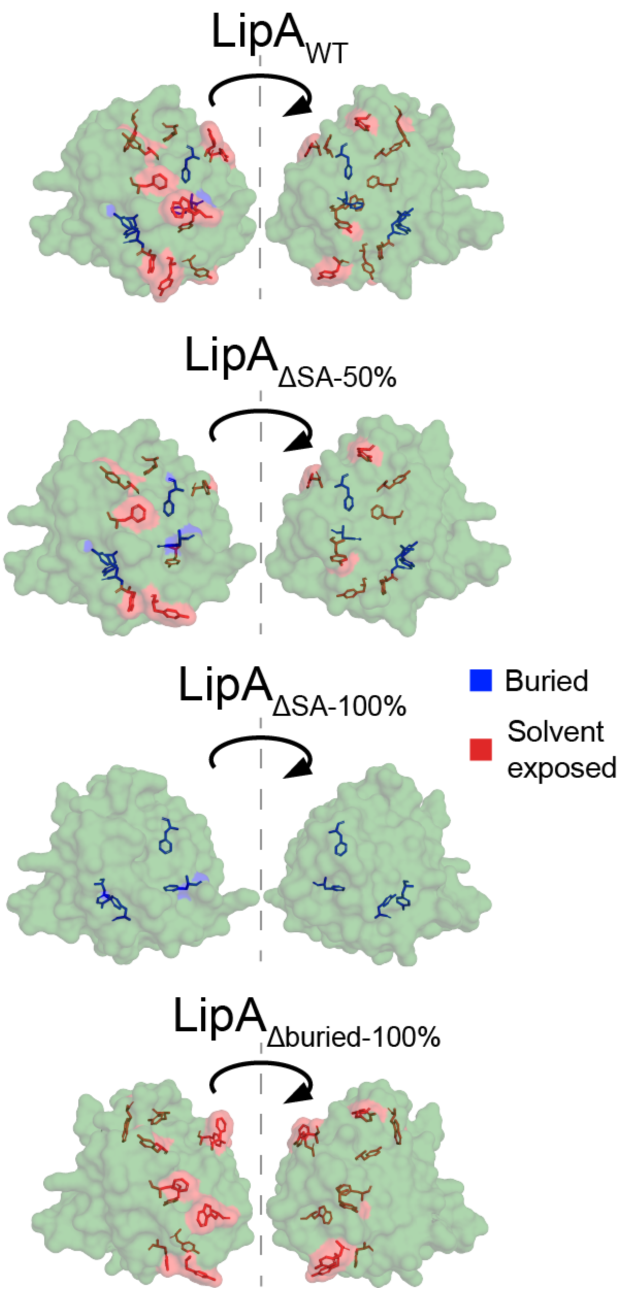

Supplementary Figure 4. Structures of LipA$_{WT}$ and Rosetta-designed mutants, where solvent-exposed aromatic residues are colored in red, and buried aromatic residues are colored in blue. Images were generated in PyMOL. The original PDB ID used for LipA was 1ISP, and the PDB files for mutants were generated by Rosetta after mutation and relaxation, as explained in the Methods section.

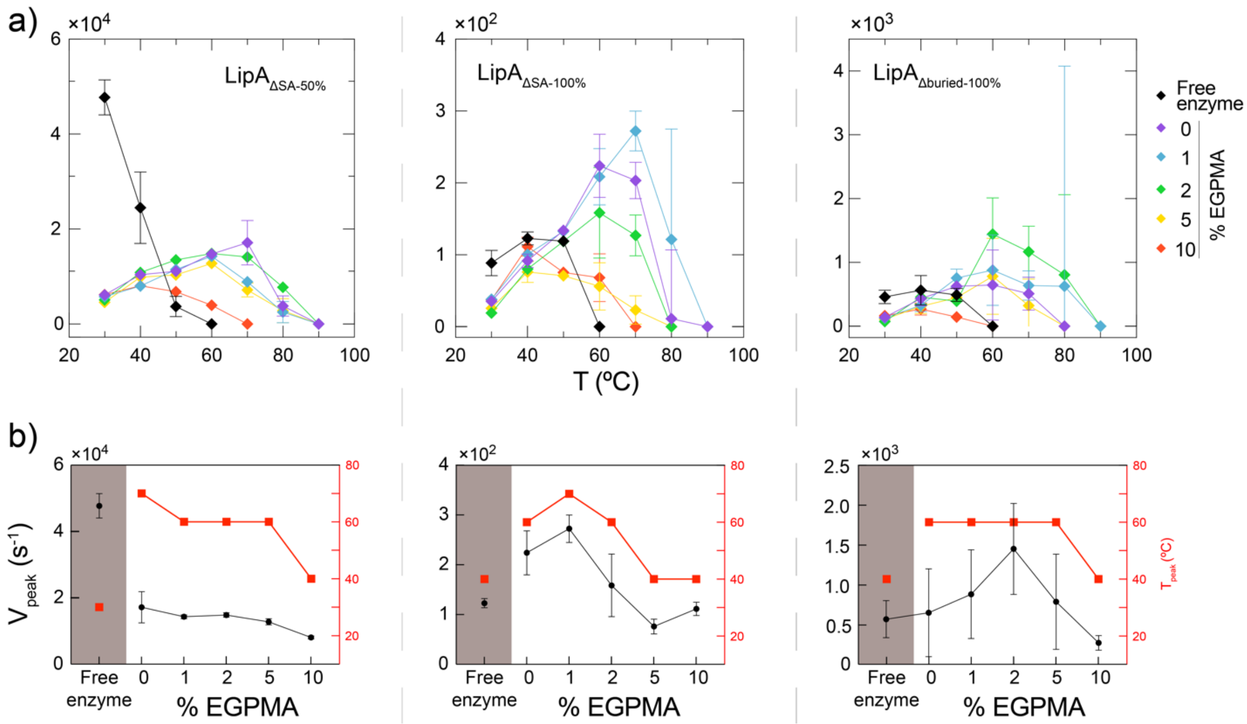

Supplementary Figure 5. **a)** Temperature-dependent activity plots of free and immobilized wild type LipA and mutants on SBMA/EGPMA supports plotted using a linear scale for apparent turnover from **Fig. 3**. Error bars represent the standard error of the mean of three technical replicates for each measurement (n = 3). **b)** Plots of V_{opt} and T_{opt} for LipA mutants for free and immobilized enzyme. Error bars represent the standard error of the mean of three technical replicates for each measurement (n = 3).

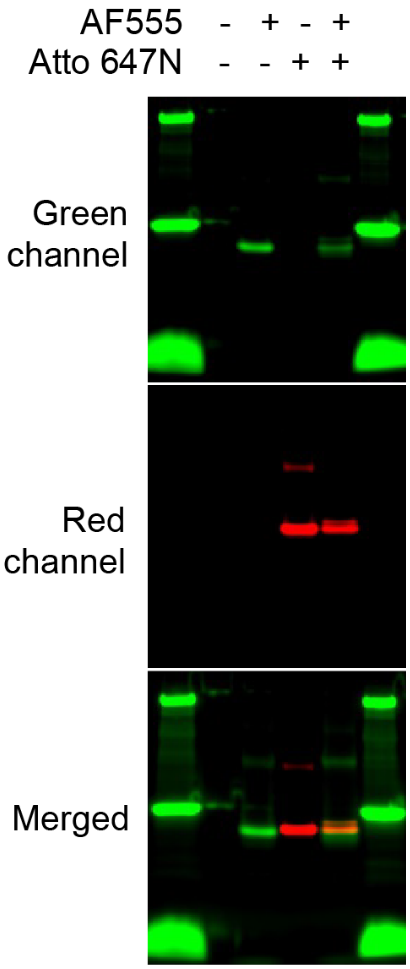

Supplementary Figure 6. SDS electrophoresis gel of LipA-FRET. Lane 1: ladder. Lane 2: Unlabeled LipA. Lane 3: AF555-labeled LipA. Lane 4: Atto647N-labeled LipA. Lane 5: dual labeled LipA-FRET. Lane 6: Ladder. Imaging was performed using a GE Typhoon FLA 9000 gel imager using Alexa Fluor 555 channel and Alexa Fluor 647 channel.

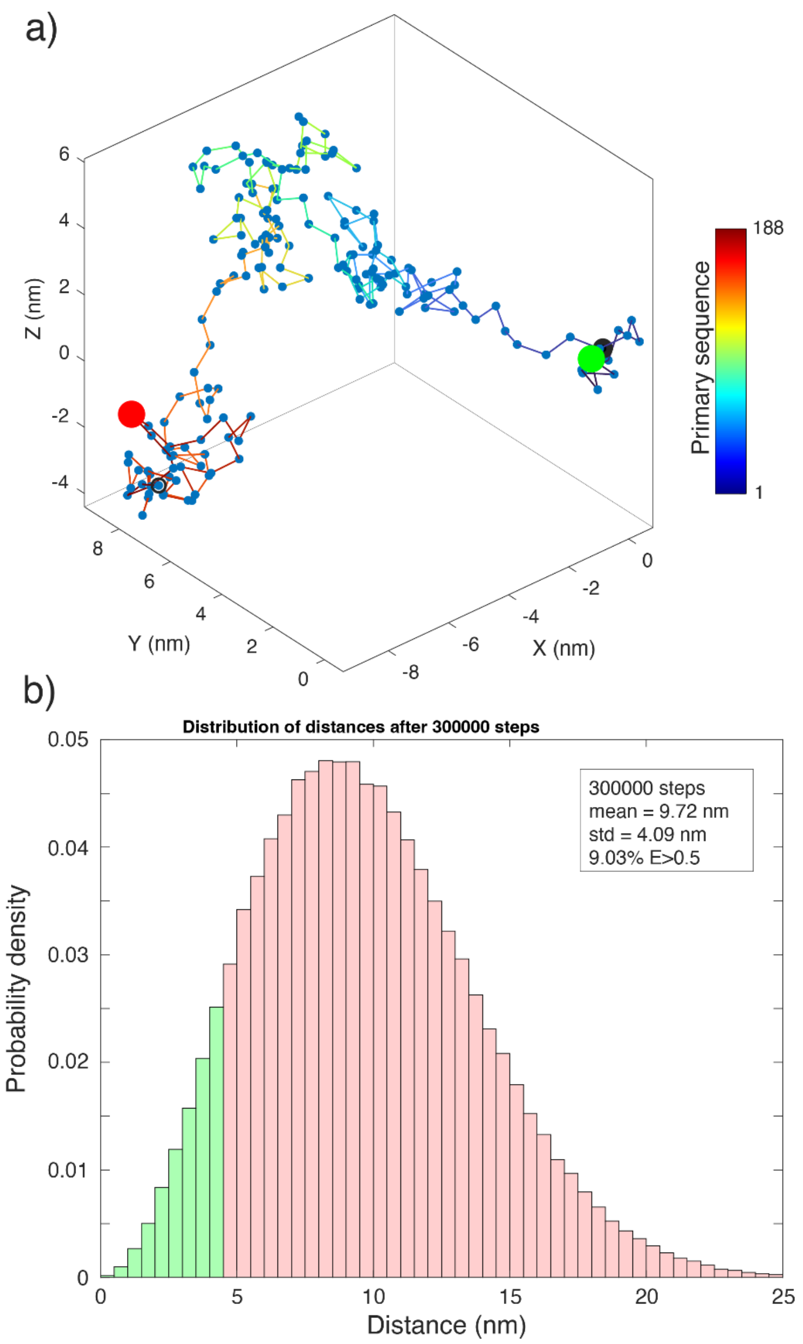

Supplementary Figure 7. **a)** Representative time step of a Monte Carlo simulation for 3D unfolded states of LipA modeled as a self-avoiding random coil, as decribed in the **Supplementary Methods**. Color code represents the primary sequence position, green and red beads represent AF555 and Atto 647N fluorophores (in positions 4 and 175, respectively), and black beads represent the start (filled) and end (unfilled) of the polypeptide chain. **b)** Inter-fluorophore distance distribution between AF555 and Atto 647N based on the Monte Carlo simulation with 300000 steps. The region in green represents the fraction of unfolded states that would yield FRET efficiency (E) > 0.5 and hence a false positive of the folded state, whereas the red region represents the fraction of the distribution that would yield E_{FRET} < 0.5 (unfolded state).

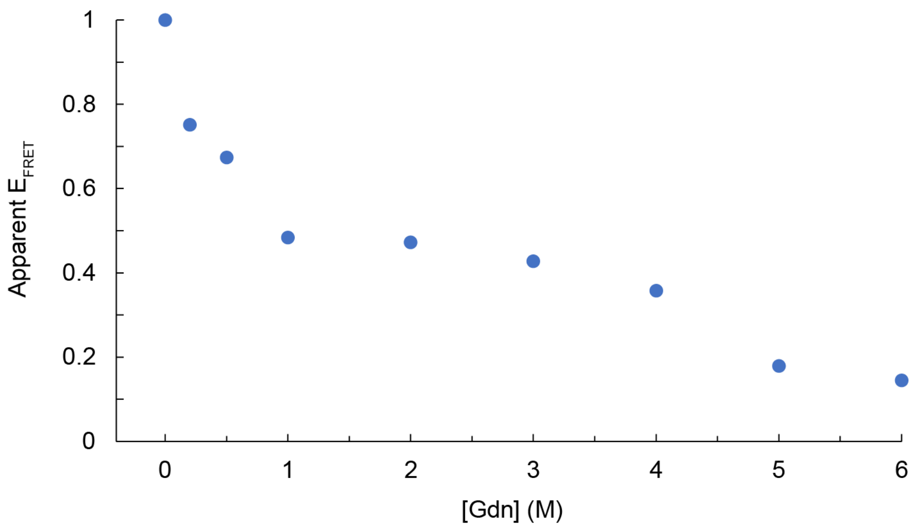

Supplementary Figure 8. Chemical denaturation curve of LipA-FRET, using guanidine hydrochloride (Gdn) as denaturant. In addition to the concentration of Gdn, all conditions had 50mM sodium phosphate at pH 7. Samples were allowed to equilibrate for 30 minutes before measurements were acquired.

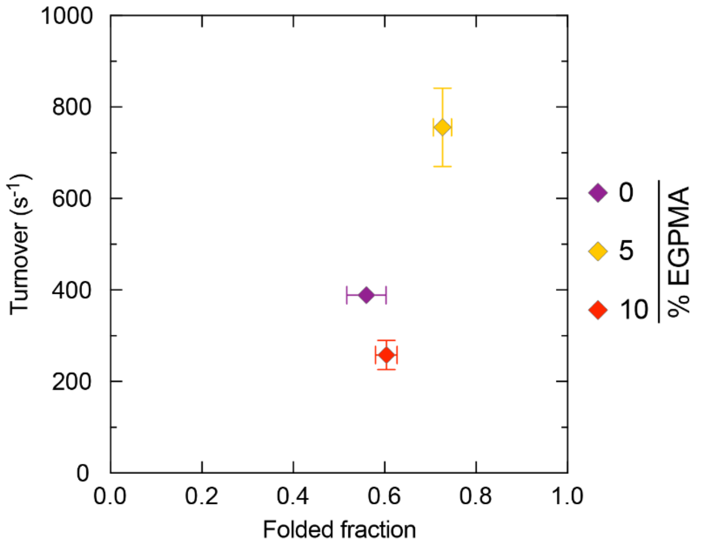

Supplementary Figure 9. Relationship between macroscopically-measured enzymatic activity and single-molecule-determined folded fraction for immobilized LipA at 45ºC. Vertical error bars represent the standard error of the mean for three technical replicates (n = 3). Horizontal error bars correspond to error in folded fraction for five iterations (n = 5) of dashed line determination from **Fig. 3**.

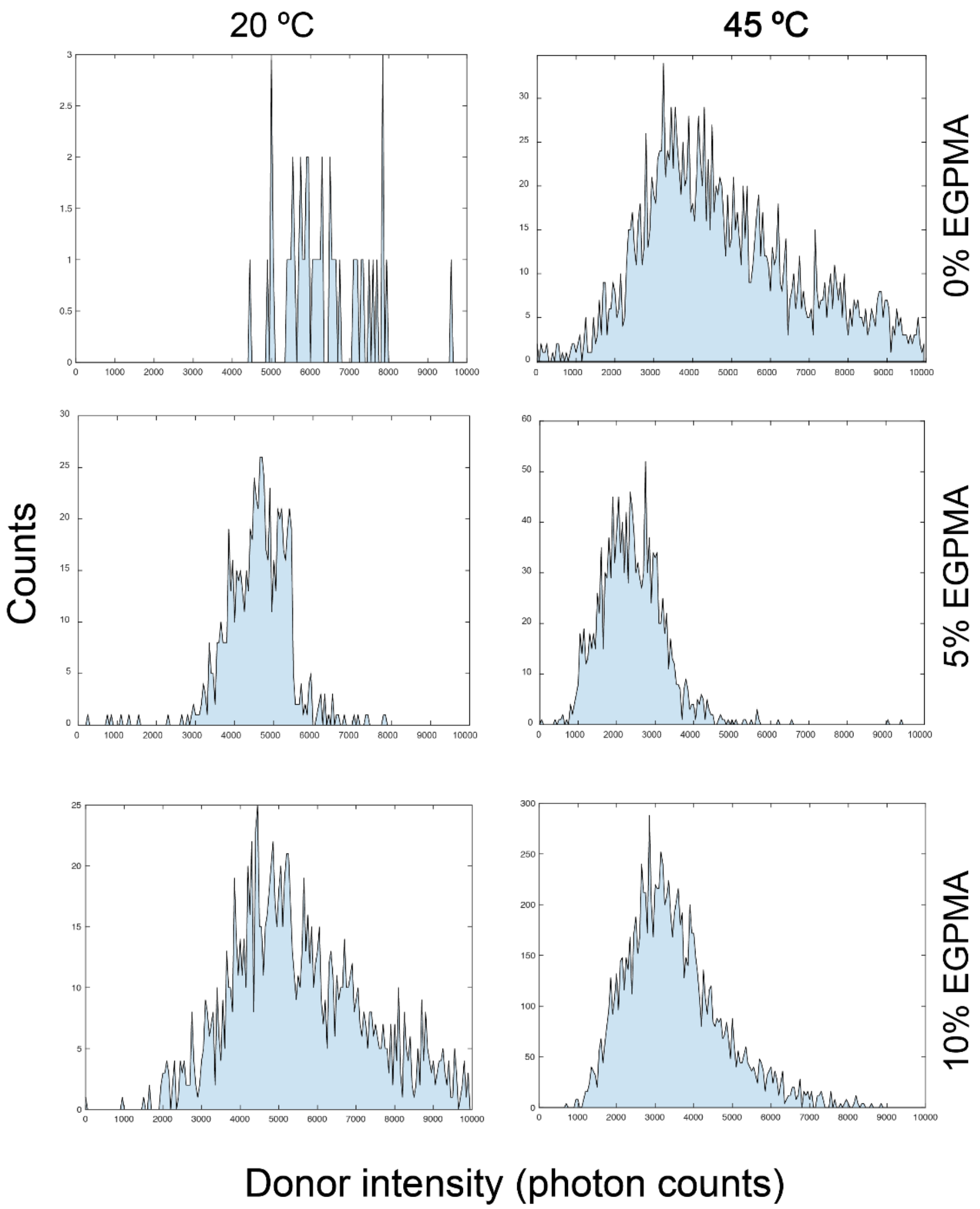

Supplementary Figure 10. Distribution of donor intensities of unfolded states from **Fig. 3b**. To generate these plots from each FRET heatmap, a window of acceptor intensities that discriminated folded observations was selected.

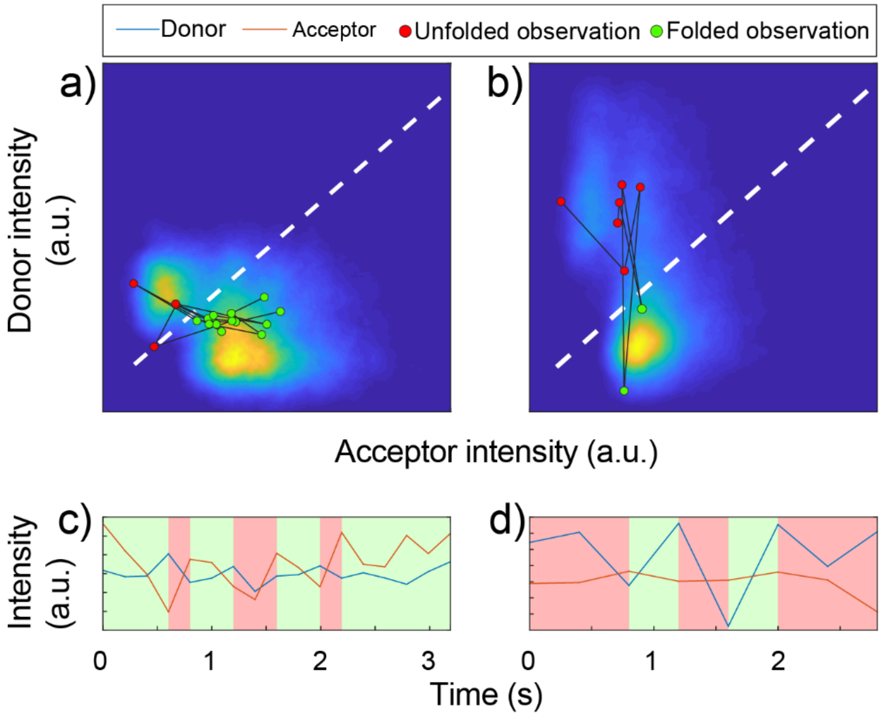

Supplementary Figure 11. Representative examples of LipA-FRET trajectories in a) 5% EGPMA at 45ºC and b) 10% EGPMA at 20ºC. Underneath, donor and acceptor intensities time series (c and d) are shown from trajectories above, where donor and acceptor intensities for each time point are overlapped in FRET heatmaps. Green and red shaded regions in panels c) and d) correspond to intervals when LipA-FRET is folded or unfolded, respectively.

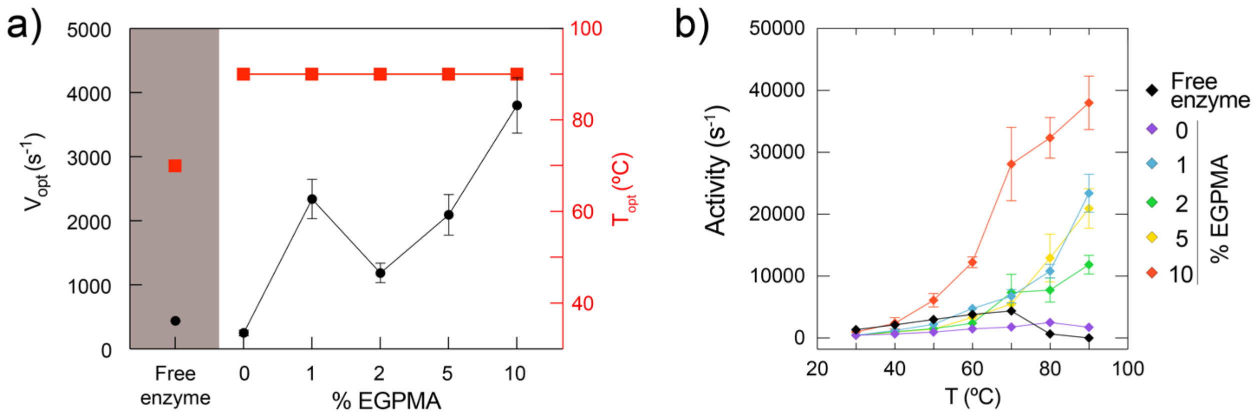

Supplementary Figure 12. Enzymatic activity of hCAII under thermally denaturing conditions. **a)** Optimal activity (V_{opt}) and temperature of optimal activity (T_{opt}) of immobilized hCAII extracted from **Fig. 6a** as a function of polymer brush composition. The data points in the shaded region correspond to V_{opt} and T_{opt} values for free enzyme. Error bars represent the standard error of the mean for three technical replicates (n = 3). **b)** Temperature-dependent activity profile for hCAII for free and immobilized enzyme. This plot represent the same data as **Fig. 6** but with enzymatic turnover in linear scale. The error bars represent the standard error of the mean for three technical replicates (n = 3).

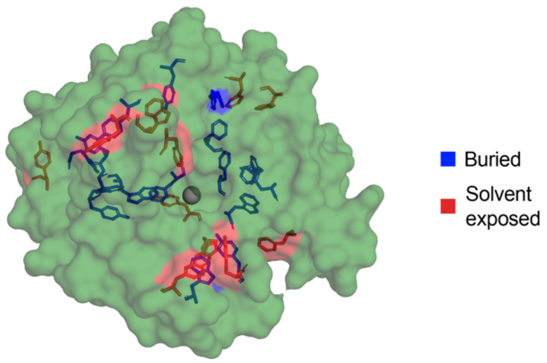

Supplementary Figure 13. Structure of hCAII, where solvent-exposed aromatic residues are colored in red, and buried aromatic residues are colored in blue, and solvent-exposed catalytic zinc is colored in gray. Image was generated in PyMOL. The PDB ID used for this image is 6B00.

```
LipA_WT              MAEHNPVVMVHGIGGASFNFAGIKSYLVSQGWSRDKLYAVDFWDKTGTNYNNGPVLSRFV   60
LipA_ΔSA-50%         .................A.............A..........A.................   60
LipA_ΔSA-100%        .................A.......A.....A.....A....A......A........A.   60
LipA_Δburied-100%    ...................M.....................M..................   60

LipA_WT              QKVLDETGAKKVDIVAHSMGGANTLYYIKNLDGGNKVANVVTLGGANRLTTGKALPGTDP  120
LipA_ΔSA-50%         ............................................................  120
LipA_ΔSA-100%        .........................A..................................  120
LipA_Δburied-100%    ..........................M.................................  120

LipA_WT              NQKILYTSIYSSADMIVMNYLSRLDGARNVQIHGVGHIGLLYSSQVNSLIKEGLNGGGQN  180
LipA_ΔSA-50%         ...................A........................................  180
LipA_ΔSA-100%        ...................A.....................A..................  180
LipA_Δburied-100%    .....V......................................................  180

LipA_WT              TNHHHHHH  188
LipA_ΔSA-50%         ........  188
LipA_ΔSA-100%        ........  188
LipA_Δburied-100%    ........  188
```

Supplementary Figure 14. Multiple sequence alignment of wild-type LipA ($LipA_{WT}$) and all designed mutants. Solvent-exposed aromatic residues are highlighted in magenta, and buried aromatic residues are highlighted in cyan. The replacement amino acids for each position and mutant are colored in red.

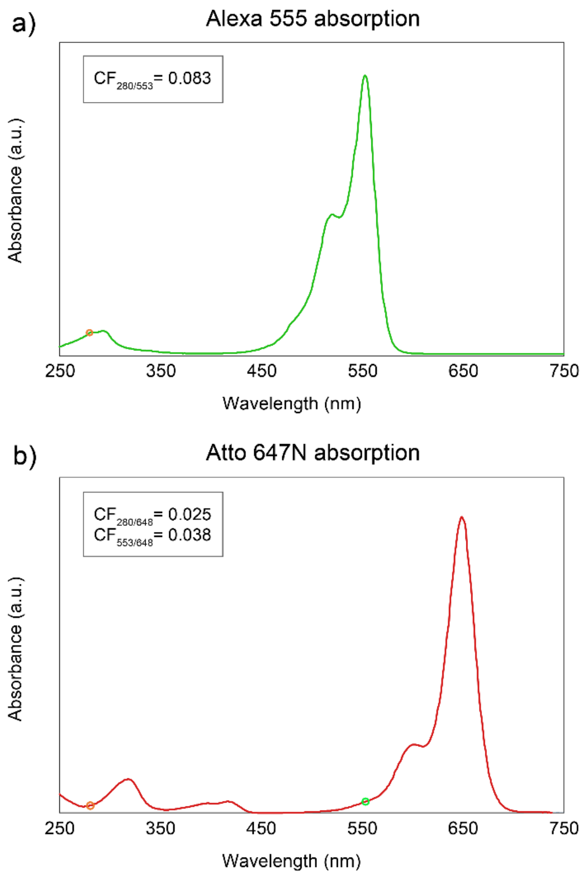

Supplementary Figure 15. Optical absorption spectra of (a) Alexa Fluor 555 and (b) Atto 647N fluorophores. In each plot, the correction factors for overlapping signals (CF) is calculated by normalizing 280 and 553 nm signals to the as the maximal absorbance of each fluorophore. In each plot, the two wavelengths at which CF's are applied are marked with a circle.

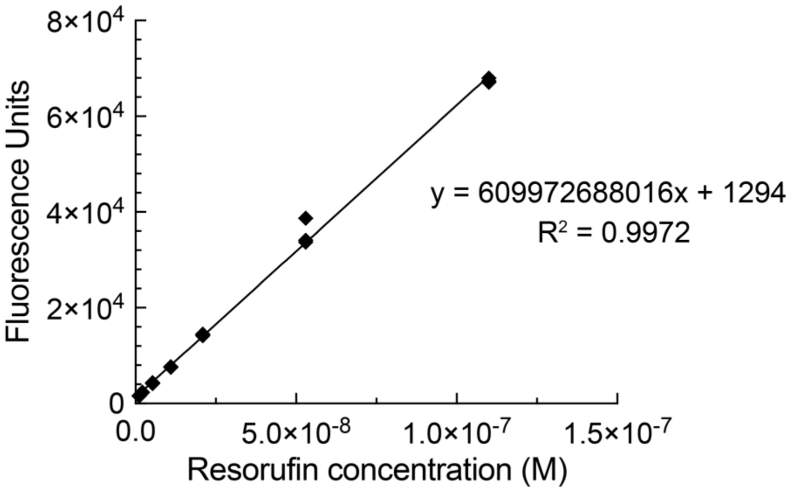

Supplementary Figure 16. Fluorescence calibration for resorufin. Three technical replicates for each resorufin concentration are represented as individual data points and the equation of linear fit was determined using GraphPad Prism.

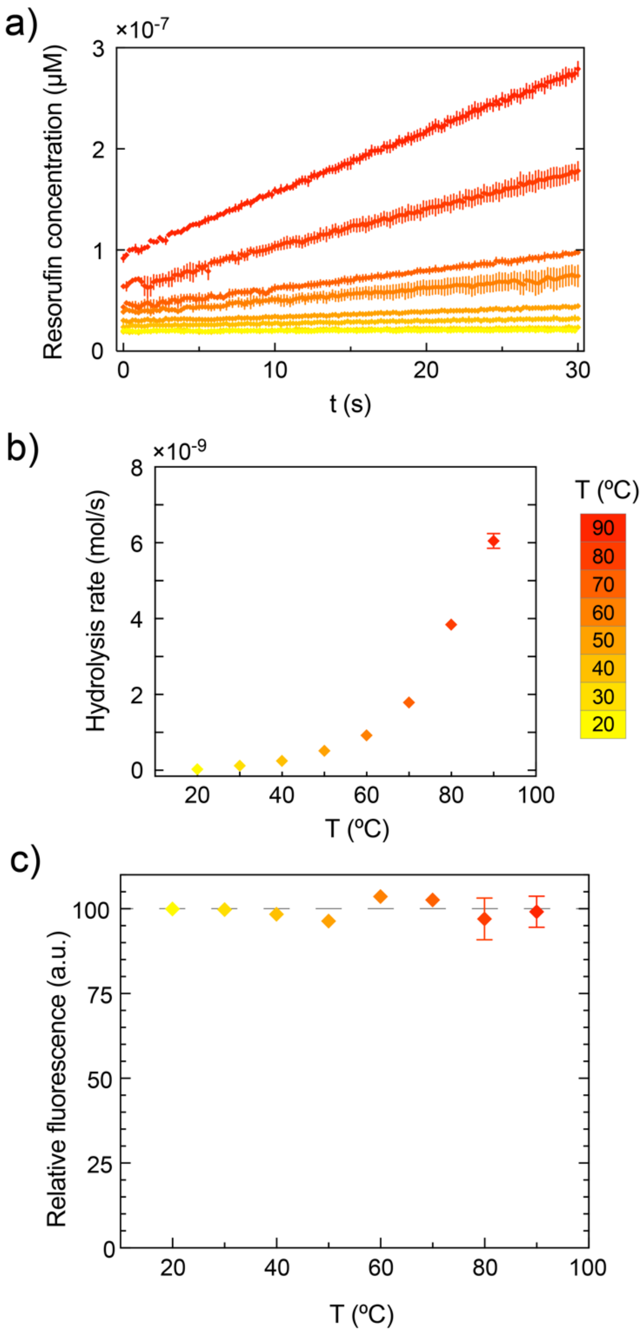

Supplementary Figure 17. Resorufin butyrate spontaneous hydrolysis at high temperatures. a) Product release profiles of resorufin butyrate as a function of temperature. Error bars correspond to the standard error of the mean for 3 technical replicates (n = 3). b) Rates of resorufin butyrate hydrolysis obtained from the slopes of data in panel a. Error bars correspond to the standard error of the mean of the slopes of each technical replicate for each temperature (n = 3). c) Relative fluorescence of 1 µM resorufin up to 90 ºC with respect to fluorescence at 20 ºC, where it can be observed that fluorescence is stable for all measured temperatures. Error bars represent the standard error of the mean for three technical replicates (n = 3).

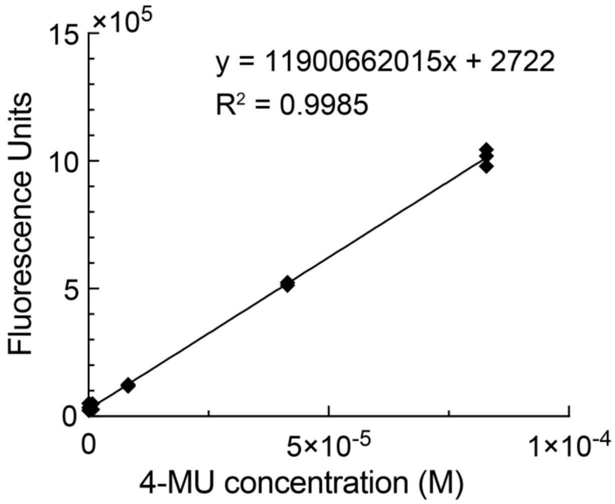

Supplementary Figure 18. Fluorescence calibration for 4-methylumbelliferone (4-MU). Three technical replicates for each 4-MU concentration are represented as individual data points and the equation of linear fit was determined using GraphPad Prism.

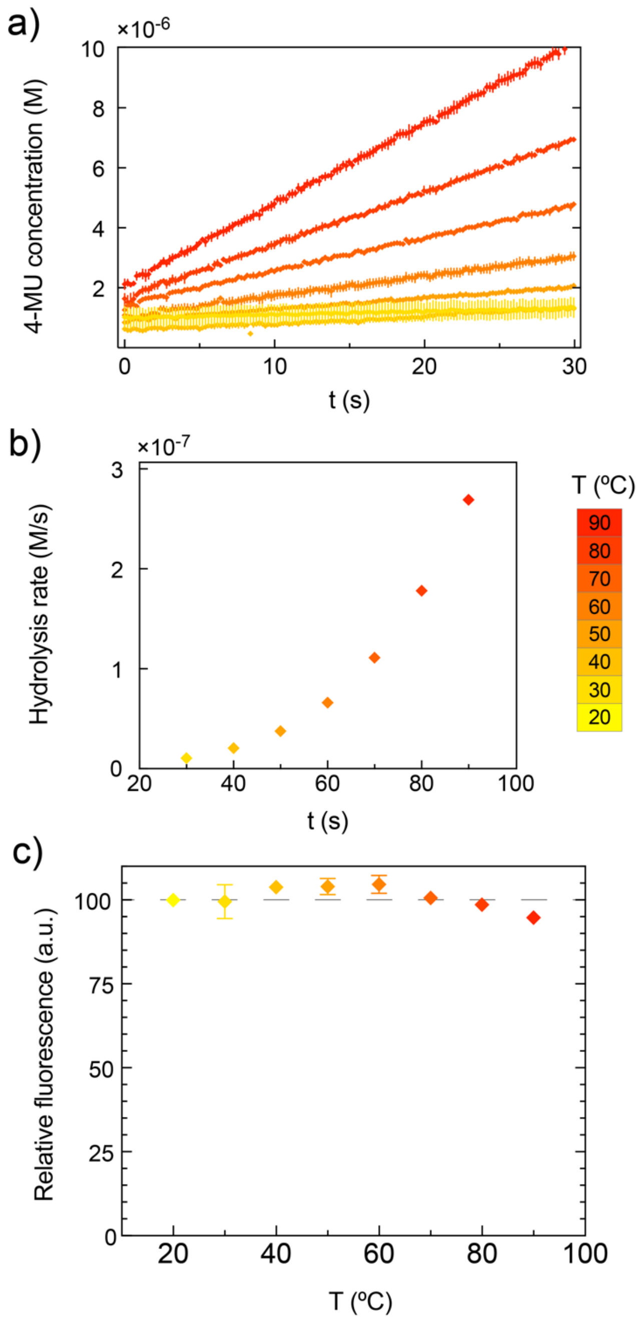

Supplementary Figure 19. 4-methylumbelliferyl acetate spontaneous hydrolysis at high temperatures. a) Product release profiles of 4-methylumbelliferyl acetate as a function of temperature. Error bars correspond to the standard error of the mean for 3 technical replicates (n = 3). b) Rates of 4-methylumbelliferyl acetate hydrolysis obtained from the slopes of panel a. Error bars correspond to the standard error of the mean of the slopes of each technical replicate for each temperature (n = 3). c) Relative fluorescence of 1 µM 4-methylumbelliferone up to 90 ºC with respect to fluorescence at 20 ºC, where it can be observed that fluorescence is stable for all measured temperatures. Error bars represent the standard error of the mean for three technical replicates (n = 3).

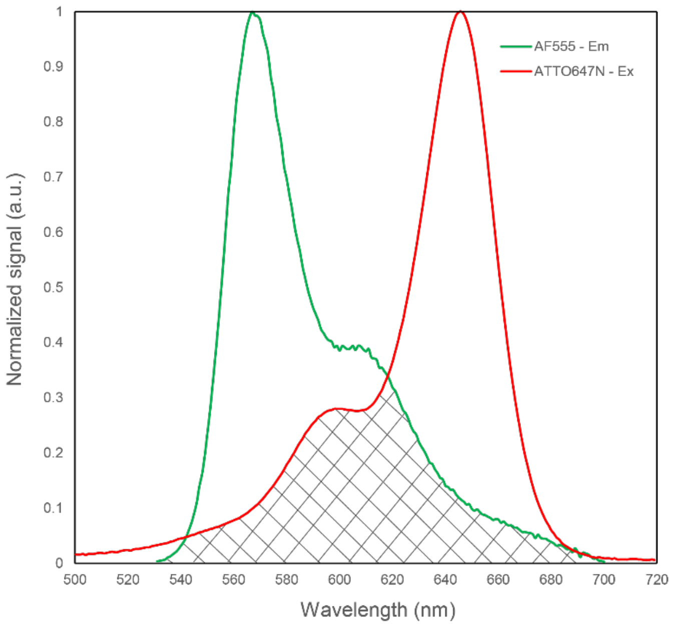

Supplementary Figure 20. Overlap region between emission spectrum of Alexa Fluor 555 and excitation spectrum of Atto 647N fluorophores. The dashed region was used for determination of the overlap integral J(λ) to quantify the Förster radius of this pair of fluorophores, as explained in the **Supplementary Methods**.

Supplementary Tables

Supplementary Table 1. Estimated reactivity of LipA residues with GMA. Residue reactivities are classified by fast, slow or non-reacting, based on their estimated protonation at pH 8.7 during immobilization and solvent accessibility. Protonation was determined using APBS server (https://server.poissonboltzmann.org/) and residue accessibility was determined using GetArea server (https://curie.utmb.edu/getarea.html).

Residue	pKa	% solvent accesibility	Reactive?
N-term	5.9	66.4	Fast-reacting
HIS-3	6.3	18.1	Non-reacting
HIS-10	5	4.7	Non-reacting
LYS-23	9.5	11.6	Non-reacting
TYR-25	11.3	25	Non-reacting
LYS-35	>12.0	34.7	Non-reacting
TYR-37	>12.0	20.1	Non-reacting
LYS-44	10.8	90.4	Non-reacting
TYR-49	10.8	71.4	Non-reacting
LYS-61	11.7	73.7	Non-reacting
LYS-69	10.8	97.3	Non-reacting
LYS-70	11.7	21.7	Non-reacting
HIS-76	2.3	6.1	Non-reacting
TYR-85	10.5	22	Non-reacting
TYR-86	>12.0	0.1	Non-reacting
LYS-88	10.2	54	Slow-reacting

Residue	pKa	% solvent accesibility	Reactive?
LYS-95	>12.0	28	Non-reacting
LYS-112	8	18.5	Non-reacting
LYS-122	3.7	10	Non-reacting
TYR-125	>12.0	0.9	Non-reacting
TYR-129	9.8	3.8	Non-reacting
TYR-139	9.9	72.9	Slow-reacting
HIS-152	6.2	71.8	Fast-reacting
HIS-156	7.6	4.5	Non-reacting
TYR-161	10.9	55.8	Non-reacting
LYS-170	>12.0	42.8	Non-reacting
HIS-182	0.3	29.9	Slow-reacting
HIS-183	4.9	56.4	Fast-reacting
HIS-184	6	82.1	Fast-reacting
HIS-185	6.1	81.8	Fast-reacting
HIS-186	5.9	54.5	Fast-reacting
HIS-187	1.8	75.4	Fast-reacting

Supplementary Table 2. Data of loaded wild-type LipA for each support used in activity assays. Moles of active site were estimated using folded fraction data from SM experiments. Folded fractions for 1% and 2% EGPMA were estimated by linear interpolation using folded fractions at 0% and 5% EGPMA.

		% EGPMA				
		0 %	1 %	2 %	5 %	10 %
$LipA_{WT}$	mg/g support	0.381	0.221	0.253	0.159	0.281
	mol active site /g support	1.75 E-05	9.75 E-06	1.07 E-05	5.90 E-06	9.53 E-06

Supplementary Table 3. Apparent Michaelis-Menten parameters of soluble and immobilized LipA at 20ºC and 45ºC. Activity as a function of resorufin butyrate concentration (10 - 50 µM, 10 µM increments) was measured and fit to a Michaelis-Menten model using non-linear regression in Matlab. Errors for K_m and k_{cat}, were obtained from the fit, and propagated for k_{cat}/K_m. Each activity measurement was acquired as three technical replicates (n = 3).

K_m ($LipA_{WT}$) (M)	20ºC	45ºC
Free enzyme	1.7×10^{-5} (4.1×10^{-6})	-
0% EGPMA	1.4×10^{-5} (3.4×10^{-6})	2.8×10^{-5} (1.5×10^{-5})
1% EGPMA	1.1×10^{-5} (2.5×10^{-6})	3.9×10^{-5} (1.0×10^{-5})
2% EGPMA	1.2×10^{-5} (1.8×10^{-6})	3.3×10^{-5} (1.6×10^{-5})
5% EGPMA	1.6×10^{-5} (5.0×10^{-6})	3.7×10^{-5} (1.6×10^{-5})
10% EGPMA	1.5×10^{-5} (3.1×10^{-6})	2.0×10^{-5} (6.1×10^{-6})

k_{cat} ($LipA_{WT}$) (s^{-1})	20ºC	45ºC
Free enzyme	2.7×10^{1} (4.2×10^{0})	-
0% EGPMA	2.7×10^{1} ($4. \times 10^{0}$)	7.0×10^{1} (1.9×10^{1})
1% EGPMA	5.9×10^{1} (9.7×10^{0})	1.7×10^{2} (2.0×10^{1})
2% EGPMA	4.1×10^{1} (4.2×10^{0})	7.2×10^{1} (1.8×10^{1})
5% EGPMA	1.1×10^{2} (2.4×10^{1})	1.9×10^{2} (3.9×10^{1})
10% EGPMA	5.2×10^{1} (7.4×10^{0})	5.3×10^{1} (9.8×10^{0})

k_{cat}/K_m ($LipA_{WT}$) (M^{-1} s^{-1})	20ºC	45ºC
Free enzyme	1.6×10^{6} (4.5×10^{5})	-
0% EGPMA	2.0×10^{6} (6.1×10^{5})	2.5×10^{6} (1.5×10^{6})
1% EGPMA	5.4×10^{6} (1.5×10^{6})	4.2×10^{6} (1.2×10^{6})
2% EGPMA	3.3×10^{6} (5.9×10^{5})	2.2×10^{6} (1.2×10^{6})
5% EGPMA	7.2×10^{6} (2.8×10^{6})	5.0×10^{6} (2.4×10^{6})
10% EGPMA	3.6×10^{6} (9.2×10^{5})	2.7×10^{6} (9.4×10^{5})

Supplementary Table 4. Features of LipA mutants used to test the effect of eliminating aromatic residues.

	**$LipA_{WT}$**	**$LipA_{\Delta SA-50\%}$**	**$LipA_{\Delta SA-100\%}$**	**$LipA_{\Delta buried-100\%}$**
Description	Native enzyme	50% exposed aromatics removed	All exposed aromatics removed	100% buried aromatics removed
% aromatic surface (hi-patch output)	7.25%	3.99%	0.8%	7.42%
ΔG^{solv}/area ($kJ/mol \cdot nm^2$)	-15.53	-15.16	-15.58	-15.63
Rosetta energy score (REU)	-546.4	-542.0	-542.0	-547.5
Ratio exposed/buried	11/5 = 2.2	7/5 = 1.4	0/5 = 0	11/1 = 11

Supplementary Table 5. Michaelis-Menten constants (K_m) for wild-type and mutants of LipA. Activity as a function of resorufin butyrate concentration (10 - 50 µM, 10 µM increments) was measured and fit to a Michaelis-Menten model using non-linear regression in Matlab. Errors for K_m was obtained from the fit. Each activity measurement was acquired as three technical replicates (n = 3).

	K_m (20 ºC)
$LipA_{WT}$	1.7×10^{-5} (4.1×10^{-6})
$LipA_{\Delta SA-50\%}$	1.4×10^{-5} (2.6×10^{-6})
$LipA_{\Delta SA-100\%}$	1.5×10^{-4} (9.5×10^{-5})
$LipA_{\Delta buried-100\%}$	2.7×10^{-5} (6.0×10^{-6})

Supplementary Table 6. Data of loaded LipA mutants for each support used in activity assays.

		% EGPMA				
		0 %	1 %	2 %	5 %	10 %
$LipA_{\Delta SA\text{-}50\%}$	mg/g support	0.706	0.668	0.589	0.633	0.666
$LipA_{\Delta SA\text{-}100\%}$	mg/g support	2.03	2.28	2.26	2.20	2.13
$LipA_{\Delta buried\text{-}100\%}$	mg/g support	0.151	0.141	0.155	0.140	0.142

Supplementary Table 7. Number of analyzed trajectories for each single molecule experiment condition. These were the trajectories remaining after applying filtering criteria described in the Methods section.

**Support Composition (% EGPMA)**	**20ºC**	**45ºC**
0%	1347	2868
5%	1878	1512
10%	3149	2771

Supplementary Table 8. Data of loaded hCAII for each support used in activity assays.

		% EGPMA				
		0 %	1 %	2 %	5 %	10 %
hCAII	mg/g support	8.60	1.70	4.23	2.56	0.810

Supplementary Table 9. Molar ratios of reagents in ARGET ATRP reactions for the synthesis of polymer brushes with different SBMA/EGPMA ratios. The ratios are normalized to the concentration of the copper catalyst. X represents the percentage of EGPMA (% EGPMA) of each polymer brush batch.

Compound	Molar ratio
SBMA	(446.5 x (100-X))/100
PEGMA	(446.5 x X)/100
Glycidyl methacrylate	23.5
L-ascorbic acid	10
MeOH	81700
DMF	18295
$CuBr_2$	1
PMDETA	10
Cu(0) wire	-

Supplementary Table 10. Genes of LipA mutants which were introduced into pET21-b(+). These genes were expressed with a C-terminal hexa-histidine tail characteristic of the expression vector.

LipA$_{\Delta SA\text{-}50\%}$	ATGGCTGAACACAATCCCGTCGTAATGGTTCACGGTATCGGTGGCGCTTCCGCAAACTTCGCCGGTATCAAGAGCTATCTGGTTAGCCAAGGTGCGAGCCGTGATAAATTATACGCGGTTGACTTCGCAGATAAGACCGGCACCAATTATAACAATGGTCCGGTTTTGTCCCGCTTTGTGCAGAAGGTGCTGGACGAAACTGGGGCTAAAAAAGTTGATATCGTTGCCCATTCGATGGGCGGTGCTAACACCCTGTACTACATCAAGAACCTGGATGGCGGTAACAAAGTCGCGAACGTGGTGACCCTCGGTGGTGCGAACCGTTTGACGACGGGCAAAGCGCTGCCGGGTACCGACCCGAATCAGAAGATCTTGTATACCTCTATTTACTCTAGCGCGGACATGATTGTCATGAACGCACTTTCGCGCTTGGACGGTGCGCGTAATGTTCAAATTCACGGCGTGGGTCATATTGGCCTGCTGTACAGCTCCCAGGTGAATAGCCTGATCAAGGAGGCCTGAACGGTGGCGGCCAAAATACCAAC
LipA$_{\Delta SA\text{-}100\%}$	ATGGCTGAACACAATCCCGTAGTCATGGTTCACGGCATCGGCGGTGCTTCTGCTAACTTTGCCGGTATTAAAAGCGCGTTGGTTAGCCAAGGTGCGAGCCGTGATAAGCTGGCGGCAGTTGACTTCGCCGATAAGACCGGTACTAACGCGAACAACGGTCCGGTTCTCAGCCGCGCAGTTCAAAAGGTCTTGGACGAGACGGGCGCGAAAAAAGTAGACATCGTGGCACATAGCATGGGTGGCGCGAACACCCTGGCGTATATCAAGAACCTGGACGGCGGAAACAAAGTGGCCAACGTGGTGACCCTGGGTGGCGCGAACCGTCTGACCACCGGTAAAGCATTACCGGGTACGGATCCGAATCAGAAGATCCTGTACACCAGCATTTACTCCTCTGCTGACATGATTGTCATGAATGCTCTTAGTCGCCTGGATGGCGCGCGTAATGTGCAGATTCATGGTGTTGGTCACATCGGTTTGCTGGCGTCGTCCCAAGTTAATAGCCTGATTAAAGAAGGTTTGAATGGCGGCGGCCAGAACACCAAC
LipA$_{\Delta buried\text{-}100\%}$	ATGGCTGAACACAATCCCGTCGTAATGGTTCACGGCATCGGCGGCGCTTCGTTCAACATGGCTGGTATCAAAAGCTATCTGGTCAGCCAAGGTTGGAGCCGCGATAAGCTGTACGCAGTTGACATGTGGGATAAGACCGGCACGAACTACAACAACGGTCCGGTTCTGTCTCGTTTTGTGCAAAAGGTCCTCGACGAAACCGGTGCGAAAAAGGTAGACATCGTGGCACATAGCATGGGTGGCGCCAACACCTTGTACATGATCAAGAACCTGGACGGCGGTAATAAAGTGGCGAACGTGGTGACTTTAGGTGGCGCGAACCGTCTGACCACCGGTAAAGCGCTGCCGGGTACGGATCCGAATCAGAAAATCCTGGTTACCAGCATTTATTCCAGCGCGGATATGATTGTGATGAATTATCTGTCCCGCTTGGACGGCGCCCGTAATGTTCAAATTCATGGTGTTGGTCATATCGGTTTGCTGTACTCCTCTCAGGTTAATAGCCTGATTAAAGAGGGCTTGAATGGCGGAGGTCAGAACACCAAC

Supplementary methods

FRET transfer efficiency and Förster radius calculation

The FRET transfer efficiency is governed by the following equation:[1]

$$E_{FRET} = \frac{1}{1 + \left(\frac{R}{R_0}\right)^6}$$

where E represents energy transfer efficiency, R represents distance between the donor and acceptor fluorophore, and R_0 denotes the Förster radius characteristic for the pair of fluorophores used. For the pair of fluorophores Alexa Fluor 555 and Atto 647N, their associated Förster radius was analytically calculated using the following equations:

$$J(\lambda) = \frac{\int_0^\infty F_D(\lambda)\, \varepsilon_A(\lambda) \lambda^4 d\lambda}{\int_0^\infty F_D(\lambda)\, d\lambda} \qquad R_0 = 0.211 \cdot \sqrt[6]{\kappa^2 n^{-4} Q_D J(\lambda)}$$

where J is the overlap integral between donor's emission and acceptor's excitation spectra (**Supplementary Fig. 20**), F_D is the peak-normalized fluorescence emission spectrum of the donor, ε_A is the molar extinction coefficient of acceptor, λ is wavelength, R_0 is the Förster radius, κ^2 is the relative orientation factor between donor and acceptor, which is taken as 2/3, assuming that their respective linkers are sufficiently long and flexible to yield free fluorophore rotation; n is the refractive index of the transfer medium and Q_D is the quantum yield of the donor. The donor-acceptor overlap integral, and parameters from this calculation are summarized as:

κ^2	2/3 (free rotation)
n	1.33 (water)
Q_D	0.1
$J(\lambda)$	$4.98 \cdot 10^{15}$
R_0 (nm)	4.59

Modeling the unfolded state of LipA via Monte Carlo simulation

The distribution of inter-fluorophore distances for the unfolded state of LipA was modeled via a Monte Carlo simulation using a 3D self-avoiding random walk model. Unfolded LipA was modeled as a chain of 188 beads of equal size with a mean diameter of 9 Å, in accordance with previously described reports of mean amino acid size accounting for the volume of backbone and side chains.[2] Even though no limitations were imposed on the flexibility of turning between beads, spatial constraints were imposed to the model (i.e., two beads cannot occupy the same space or collide). Interactions between beads were not considered. For each simulation step, the distance between the two amino acids where fluorophores are site-specifically labeled (pAzF4 and C175) were computed and plotted as a histogram in **Supplementary Fig. 7**. Since the Förster radius for AF555 and Atto 647N was calculated as 4.59 nm, the density of histogram bins up to 4.5 nm was considered to estimate rate of "false positives" which could yield FRET efficiency (E) > 0.5 while unfolded. The simulation allowed us to estimate that 9% of all the possible random-coil unfolded conformations could result in E > 0.5.

Supplementary references

1 Wu, P. G. & Brand, L. Resonance Energy Transfer: Methods and Applications. *Analytical Biochemistry* **218**, 1-13 (1994).

2 Ching, C. B., Hidajat, K. & Uddin, M. S. Evaluation of Equilibrium and Kinetic Parameters of Smaller Molecular Size Amino Acids on KX Zeolite Crystals via Liquid Chromatographic Techniques. *Separation Science and Technology* **24**, 581-597 (1989).
